# Primitive Genepools of Asian Pears and Their Complex Hybrid Origins Inferred from Fluorescent Sequence-Specific Amplification Polymorphism (SSAP) Markers Based on LTR Retrotransposons

**DOI:** 10.1371/journal.pone.0149192

**Published:** 2016-02-12

**Authors:** Shuang Jiang, Xiaoyan Zheng, Peiyuan Yu, Xiaoyan Yue, Maqsood Ahmed, Danying Cai, Yuanwen Teng

**Affiliations:** 1 Department of Horticulture, The State Agricultural Ministry Key Laboratory of Horticultural Plant Growth, Development and Quality Improvement, Zhejiang University, Hangzhou, Zhejiang 310058, China; 2 Forest & Fruit Tree Institute, Shanghai Academy of Agricultural Sciences, Shanghai, 201403, China; 3 Institute of Horticulture and Landscape, College of Ecology, Lishui University, Lishui, Zhejiang 323000, China; 4 Department of Biotechnology, Mirpur University of Science and Technology (MUST), Main Allama Iqbal Road, Mirpur, Azad Kashmir-10250, Pakistan; 5 Institute of Horticulture, Zhejiang Academy of Agricultural Sciences, Hangzhou, Zhejiang Province, China; NARO Institute of Fruit Tree Science, JAPAN

## Abstract

Recent evidence indicated that interspecific hybridization was the major mode of evolution in *Pyrus*. The genetic relationships and origins of the Asian pear are still unclear because of frequent hybrid events, fast radial evolution, and lack of informative data. Here, we developed fluorescent sequence-specific amplification polymorphism (SSAP) markers with lots of informative sites and high polymorphism to analyze the population structure among 93 pear accessions, including nearly all species native to Asia. Results of a population structure analysis indicated that nearly all Asian pear species experienced hybridization, and originated from five primitive genepools. Four genepools corresponded to four primary Asian species: *P*. *betulaefolia*, *P*. *pashia*, *P*. *pyrifolia*, and *P*. *ussuriensis*. However, cultivars of *P*. *ussuriensis* were not monophyletic and introgression occurred from *P*. *pyrifolia*. The specific genepool detected in putative hybrids between occidental and oriental pears might be from occidental pears. The remaining species, including *P*. *calleryana*, *P*. *xerophila*, *P*. *sinkiangensis*, *P*. *phaeocarpa*, *P*. *hondoensis*, and *P*. *hopeiensis* in Asia, were inferred to be of hybrid origins and their possible genepools were identified. This study will be of great help for understanding the origin and evolution of Asian pears.

## Introduction

The genus *Pyrus*, with the common name pear, is believed to have originated in the mountainous areas of western and southwestern China [1]. Based on their geographic distribution, *Pyrus* is divided into two groups: occidental and oriental pears [1,2]. The oriental pears (also referred to as Asian pears) include 12–15 species, the majority being native to China [3]. Wild pea pears: *P*. *betulaefolia* Bunge and *P*. *calleryana* Decne. (including *P*. *dimorphophylla* Makino, *P*. *fauriei* C.K.Schneid., and *P*. *koehnei* C.K.Schneid., which were once classified as varieties of *P*. *calleryana*), bearing small fruit with a diameter of ~1 cm and two carpels, are believed to be ancestral species [4]. *P*. *pashia* D. Don is naturally distributed in Southwest China, and shows morphological diversity and carpel numbers from two to five [5,6]. The majority of remaining species, such as *P*. *phaeocarpa* Rehder, *P*. *hopeiensis* T.T.Yu, *P*. *xerophila* T.T.Yu and *P*. *serrulata* Rehder, have been reported to be inter-specific hybrids [7,8], but their origins were uncertain. Most cultivated pears native to Asia are assigned to three species: *P*. *ussuriensis* Maxim. (Ussurian pear), *P*. *sinkiangensis* T.T.Yu (Xinjiang pear) and *P*. *pyrifolia* Nakai [9]. Based on the geographic distribution, the cultivated *P*. *pyrifolia* was further divided into three cultivar groups: Chinese white pear group (sometimes mistakenly assigned as *P*. *bretschneideri* Rehd.), Chinese sand pear group and Japanese pear group [3]. The origin of some cultivated pear groups is still controversial. Understanding the domestication process of the cultivated pear and the evolutionary process of the pear species will be helpful in exploiting elite genetic resources in pears and aid in modern breeding.

Frequent interspecific hybridization events, which cause reticulate evolution, hamper our ability to understand the evolutionary history of the plants. Owing to the lack of a reproductive barrier among *Pyrus* species, interspecific hybridization was determined to be the major mode of *Pyrus* evolution [6,7,10,11]. DNA-based markers, such as restriction fragment length polymorphisms [12], random amplified polymorphic DNAs (RAPDs) [13], simple sequence repeats (SSRs) [14,15], and amplified fragment length polymorphisms (AFLPs) [16], have been used to analyze the genetic relationships in *Pyrus*, and some possible hybridization events that occurred in Asian pears have been identified. Recently, DNA sequences of chloroplast regions and nuclear genes were used in phylogenetic analyses of *Pyrus* [6,11,17,18]. Because in these studies, the use of cpDNA fragments and nuclear genes provides limited information sites for analysis, the details of origins of cultivated Asian pears and natural hybrid species have not been resolved.

Retrotransposons are a widespread class of transposable elements that exist in all plant species investigated to date [19–21]. They undergo replicative transposition, by way of an RNA intermediate, and thus their copy numbers increase, occupying large fractions of the genome, especially in higher plants [22–24]. Recently, retrotransposon-based markers, such as retrotransposon-based insertion polymorphisms (RBIPs) [25] and sequence-specific amplification polymorphisms (SSAPs) [26] were developed to study genetic diversity and cultivar identification. Long terminal repeat (LTR) retrotransposons are ubiquitously distributed throughout the *Pyrus* genome, and 42.4% of the genome has been reported to be LTR retrotransposons [27], implying that retrotransposons may play important roles in *Pyrus* evolution.

In previous studies, the high heterogeneity and insertion polymorphisms of retrotransposons within *Pyrus* species were shown by RBIP markers [28,29], indicating that retrotransposons replicated many times in the pear genome. However, RBIP markers only provided a few information sites. Although the use of a larger number of RBIP markers could overcome this short-coming, it would be costly. SSAP markers based on retrotransposons are similar to AFLPs [26]. They both reflect restriction size variation in the whole genome, but SSAP markers also identify polymorphisms produced by retrotransposon insertions [30]. Therefore, SSAP markers have the advantages of high heterogeneity and adequate information sites, which should make them more effective in revealing the genetic relationships within species. In the last years, SSAP markers have been ignored, with only a few studies on SSAPs reported [31,32], and efforts to develop plant SSAP markers were restricted by the lack of known LTR sequences. Recently, the whole genome of the Chinese white pear cultivar ‘Dangshansuli’ was released [27], leading to a faster and more economical approach to predicting retrotransposons and developing SSAP markers in *Pyrus*.

In this study, we developed SSAP markers based on retrotransposons across the whole pear genome to analyze *Pyrus* accessions, including cultivars/landraces and wild species from China, Japan, Korea, and Pakistan. The aim of this study was to evaluate the population structures and genetic relationships of pear species and cultivars from Asia, and to reveal the origins of Asian pears.

## Materials and Methods

### Plant materials and DNA extraction

We analyzed 93 *Pyrus* accessions from four Asian countries. Accessions native to Asia were collected from the China Pear Germplasm Repository, Xingcheng, Liaoning Province, China; Gansu Pomology Institute, Gansu Academy of Agricultural Sciences, Lanzhou, Gansu Province, China; Wuhan Sand pear Germplasm Repository, Wuhan, Hubei Province, China; Tottori University, Tottori, Japan and University of Azad Jammu and Kashmir, Azad Jammu and Kashmir, Pakistan (Table 1). Three accessions of occidental pears were collected from the National Clonal Germplasm Repository, Corvallis, OR, USA.

**Table 1:** Accessions of investigated species and cultivars of Asian pears and outgroup.

Code	Taxa	Accession	Origin	Source orLatitude (°N)/ Longitude (°E)
Oriental pear				
Chinese white pear group (CWPG)				
1	*P*. *pyrifolia*	Piaobali	Guizhou, China	CPGR
2	*P*. *pyrifolia*	Dadongguo	Gansu, China	GPI
3	*P*. *pyrifolia*	Suli	Anhui, China	CPGR
4	*P*. *pyrifolia*	Fenhongxiao	Hebei, China	CPGR
5	*P*. *pyrifolia*	Fengxianjitui	Shanxi, China	CPGR
6	*P*. *pyrifolia*	Huangjitui	Jiangsu, China	CPGR
7	*P*. *pyrifolia*	Jingchuan	Gansu, China	CPGR
8	*P*. *pyrifolia*	Xiangchun	Shanxi, China	CPGR
9	*P*. *pyrifolia*	Xuehua	Hebei, China	CPGR
10	*P*. *pyrifolia*	Yali	Hebei, China	TU
11	*P*. *pyrifolia*	Yinbai	Hebei, China	CPGR
12	*P*. *pyrifolia*	Xiaojin	Shaanxi, China	CPGR
13	*P*. *pyrifolia*	Dongguo	Gansu, China	GPI
14	*P*. *pyrifolia*	Daaoao	Shangdong, China	CPGR
15	*P*. *pyrifolia*	Eli	Liaoning, China	CPGR
16	*P*. *pyrifolia*	Zhimasu	Hubei, China	CPGR
17	*P*. *pyrifolia*	Qixiaxiaoxiangshui	Shandong, China	CPGR
Chinese sand pear group				
18	*P*. *pyrifolia*	Baozhuli	Yunnan, China	WSGR
19	*P*. *pyrifolia*	Hongshaobang	Sichuan, China	WSGR
20	*P*. *pyrifolia*	Mandingxueli	Fujian, China	WSGR
21	*P*. *pyrifolia*	Yunlu	Zhejiang, China	WSGR
22	*P*. *pyrifolia*	Baihuli	Fujian, China	WSGR
23	*P*. *pyrifolia*	*P*. *pyrifolia 1*	Yunnan, China	Yunnan, China
24	*P*. *pyrifolia*	Henshanli	Taiwan, China	WSGR
25	*P*. *pyrifolia*	Shexiangli	Hunan, China	WSGR
26	*P*. *pyrifolia*	Mashanshali	Guangxi, China	WSGR
Japanese pear group				
27	*P*. *pyrifolia*	Tosanashi	Japan	TU
28	*P*. *pyrifolia*	Kansaiyichi	Japan	TU
29	*P*. *pyrifolia*	Nekogoroshi	Japan	TU
30	*P*. *pyrifolia*	Hatsushimo	Japan	TU
31	*P*. *pyrifolia*	Tsukatanashi	Japan	TU
Accessions of *P*. *pyrifolia* from Korea				
32	*P*. *pyrifolia*	Chousennashi	North Korea	TU
33	*P*. *pyrifolia*	Hoeryongbae	North Korea	TU
34	*P*. *pyrifolia*	Hanheungli-Kou	North Korea	TU
35	*P*. *pyrifolia*	Hanheungli-Otsu	North Korea	TU
Accessions of *P*. *ussuriensis*				
36	*P*. *ussuriensis*	Hongbalixiang	Liaoning, China	CPGR
37	*P*. *ussuriensis*	Balixiang	Liaoning, China	CPGR
38	*P*. *ussuriensis*	Mangyuanxiang	Liaoning, China	CPGR
39	*P*. *ussuriensis*	Yaguangli	Hebei, China	CPGR
40	*P*. *ussuriensis*	Hongnanguoli	Liaoning, China	CPGR
41	*P*. *ussuriensis*	Nanguoli	Liaoning, China	CPGR
42	*P*. *ussuriensis*	Saozhoumiaozi	Hebei, China	CPGR
43	*P*. *ussuriensis*	Jianbali	Liaoning, China	CPGR
44	*P*. *ussuriensis*	Reqiuzi	Liaoning, China	CPGR
45	*P*. *ussuriensis*	Xiehuatian	Jilin, China	CPGR
46	*P*. *ussuriensis*	Huagai	Liaoning, China	CPGR
47	*P*. *ussuriensis*	Tianqiuzi	Liaoning, China	CPGR
48	*P*. *ussuriensis*	Ruanerli	Gansu, China	CPGR
Accessions of *P*. *sinkiangensis*				
49	*P*. *sinkiangensis*	Korlaxiangli	Xinjiang, China	CPGR
50	*P*. *sinkiangensis*	Kucheamute	Xinjiang, China	CPGR
51	*P*. *sinkiangensis*	Kunqieke	Xinjiang, China	CPGR
52	*P*. *sinkiangensis*	Zaoshujuju	Xinjiang, China	CPGR
53	*P*. *sinkiangensis*	Hesejuju	Xinjiang, China	CPGR
54	*P*. *sinkiangensis*	Ruantaijuju	Xinjiang, China	CPGR
Accessions originated from Pakistan				
55	unknown	Frashishi	Rawalakot, Pakistan	AJK
56	unknown	Nakh	Rawalakot Pakistan	AJK
57	unknown	Btangi	Rawalakot, Pakistan	AJK
58	unknown	Nashpati	Rawalakot, Pakistan	AJK
59	unknown	Bagugosha	Hajira, Pakistan	AJK
Wild pear species native to East Asia				
60	*P*. *phaeocarpa*	*P*. *phaeocarpa*	Gansu, China	GPI
61	*P*. *hondoensis*	*P*. *hondoensis*	Middle Japan	TU
62	*P*. *hopeiensis*	*P*. *hopeiensis*	Hebei, China	CPGR
63	*P*. *pashia*	*P*. *pashia 1*	Yunnan, China	25.12, 101.38
64	*P*. *pashia*	*P*. *pashia 2*	Yunnan, China	25.37, 100.85
65	*P*. *pashia*	*P*. *pashia 3*	Yunnan, China	24.97, 102.17
66	*P*. *pashia*	*P*. *pashia 4*	Yunnan, China	24.97, 102.30
67	*P*. *pashia*	*P*. *pashia 5*	Yunnan, China	24.83, 103.45
68	*P*. *pashia*	*P*. *pashia 6*	Yunnan, China	26.86, 100.16
69	*P*. *pashia*	*P*. *pashia 7*	Yunnan, China	22.45, 100.00
70	*P*. *pashia*	*P*. *pashia 8*	Yunnan, China	28.05, 99.51
71	*P*. *betulaefolia*	*P*. *betulaefolia 1*	Henan, China	33.31, 113.47
72	*P*. *betulaefolia*	*P*. *betulaefolia 2*	Shaanxi, China	36.49, 109.61
73	*P*. *betulaefolia*	*P*. *betulaefolia 3*	Shaanxi, China	35.10, 107.99
74	*P*. *betulaefolia*	*P*. *betulaefolia 4*	Hebei, China	36.51, 113.57
75	*P*. *betulaefolia*	*P*. *betulaefolia 5*	Shandong, China	35.56, 117.46
76	*P*. *betulaefolia*	*P*. *betulaefolia 6*	Shandong, China	36.22, 120.45
77	*P*. *betulaefolia*	*P*. *betulaefolia 7*	Hebei, China	36.51, 113.57
78	*P*. *betulaefolia*	*P*. *betulaefolia 8*	Gansu, China	35.45, 107.95
79	*P*. *calleryana*	*P*. *calleryana 1*	Zhejiang, China	28.78, 119.87
80	*P*. *calleryana*	*P*. *calleryana 2*	Zhejiang, China	28.78, 119.87
81	*P*. *calleryana*	*P*. *calleryana 3*	Zhejiang, China	28.78, 119.87
82	*P*. *calleryana*	*P*. *calleryana 4*	Zhejiang, China	29.87, 119.67
83	*P*. *calleryana*	*P*. *calleryana 5*	Zhejiang, China	29.87, 119.67
84	*P*. *calleryana*	*P*. *calleryana 6*	Zhejiang, China	29.87, 119.67
85	*P*. *calleryana*	*P*. *calleryana 7*	Zhejiang, China	29.53, 120.97
86	*P*. *xerophila*	*P*. *xerophila 1*	Gansu, China	GPI
87	*P*. *xerophila*	*P*. *xerophila 2*	Gansu, China	GPI
88	*P*. *xerophila*	*P*. *xerophila 3*	Gansu, China	GPI
89	*P*. *xerophila*	*P*. *xerophila 4*	Gansu, China	GPI
90	*P*. *xerophila*	*P*. *xerophila 5*	Gansu, China	GPI
Occidental pear species				
91	*P*. *gharbiana*	*P*. *gharbiana 789*	Morocco	NCGR, PI541663
92	*P*. *cordata*	*P*. *cordata 750*	France	NCGR, PI541580
93	*P*. *salicifolia*	*P*. *salicifolia 2720*	Russia	NCGR, CPYR2720

CPGR: China Pear Germplasm Repository, Xingcheng, Liaoning Province, China; GPI: Gansu Pomology Institute, Gansu Academy of Agricultural Sciences, Lanzhou, Gansu Province, China; NCGR: National Clonal Germplasm Repository, USA; TU: Tottori University, Japan; WSGR: Wuhan Sand Pear Germplasm Repository, Wuhan, Hubei Province, China; and AJK: University of Azad Jammu and Kashmir, Azad Jammu and Kashmir, Pakistan.

Total genomic DNA was extracted from the leaf tissues of plants following the modified CTAB protocol described by Doyle and Doyle [33]. The DNA concentrations were diluted to 10–30 ng μL^-1^ after the quality and quantity were determined on 1% (w v^-1^) agarose gels using standard DNA markers (Takara, Dalian, China).

### Development of SSAP markers

In a previous study, 10 subfamilies (KF806690-KF806699) of retrotransposons were isolated in *Pyrus* [29]. The conserved LTRs of these retrotransposons were used to design the primers.

### SSAP analysis

The SSAP analysis was performed using the protocol of Syed and Flavell [34]. Genomic DNA (500 ng) was digested with two restriction enzymes (EcoRI and MseI; New England Biolabs, Ipswich, MA, USA) then ligated to EcoRI/MseI adapters with T4 DNA ligase (Takara, Dalian, China). After ligation, pre-amplification was performed with EcoRI and MseI primers containing non-selective nucleotides. The pre-amplification products were diluted (1: 10) with TE buffer.

The selective amplifications were carried out with a combination of one LTR primer and one adapter-specific primer. An economic method for fluorescent labeling of PCR fragments was used during selective amplification [35]. We found 12 primer combinations that were polymorphic in our preliminary data analysis, and these were employed for the main data set analysis (Table 2). A tail (M13 universal sequence, TGTAAAACGACGGCCAGT) was added to the 5′ end of each of the MseI adapter-specific primers containing selective nucleotides (Table 2). The tail primers were labeled with the following four dyes: FAM (blue), HEX (green), NED (yellow), and PET (red). The FAM-tail and HEX-tail were synthesized by Invitrogen Trading Co., Ltd. (Shanghai, China), and the NED-tail and PET-tail by Applied Biosystems (Foster City, CA, USA). PCR amplifications were performed in a final volume of 20 μL (2 μL 10× PCR buffer, 200 μM dNTPs, 8 pmol tail primer, 2 pmol adapter-specific primer, 10 pmol LTR-specific primer, 1 U rTaq Polymerase, and 1 μL pre-amplified DNA) using the following parameters: 94°C for 5 min for initial denaturation, then 12 cycles at 94°C (30 s)/65°C (1 min)/72°C (1 min), in which the annealing temperature decreased 0.7°C per cycle, followed by 24 cycles of 94°C (30 s)/53°C (1 min)/72°C (1 min), and a final extension step of 10 min at 72°C. Amplicons were pooled together with an internal size standard (GeneScan^™^ 500 LIZ, Applied Biosystems, Foster City, CA, USA) according to the distinct dyes, and then, subsequently separated and sequenced using an ABI 3700XL Genetic Analyzer (Applied Biosystems). Fragment sizes for each accession using each primer combination were analyzed.

**Table 2:** List of primer sequences used in this study.

Primer name	Forward sequence	Reverse sequence
1	Ppcr3F: AATCTTGTATGTTGGTGGAATC	tail-M-ACT / tail
2	Ppcr1F: TGGACTTTAGATTGGGTTGTGG	tail-M-ACT / tail
3	Ppgr1F: CAATGTTGTGGCAGGTATTCA	tail-M-ACC / tail
4	Ppgr1F: CAATGTTGTGGCAGGTATTCA	tail-M-AGC / tail
5	Ppcr2F: CCAGCATTTTCAACATTACCA	tail-M-AAG / tail
6	ppgr4F: CTAGCGAAGGTCACAAACTTGA	tail-M-ACC / tail
7	Ppcr3F: AATCTTGTATGTTGGTGGAATC	tail-M-AAT / tail
8	ppgr4F: CTAGCGAAGGTCACAAACTTGA	tail-M-AGG / tail
9	Ppcr3F: AATCTTGTATGTTGGTGGAATC	tail-M-AAC / tail
10	Ppcr1F: TGGACTTTAGATTGGGTTGTGG	tail-M-AGG / tail
11	Ppcr4F: CTTGTTGCTTCCCTCCTTTCT	tail-M-AAT / tail
12	Ppcr3F: AATCTTGTATGTTGGTGGAATC	tail-M-ACG / tail

Tail indicates the M13 primer (5′TGTAAAACGACGGCCAGT 3′); M indicates the MseI adaptor (5′GATGAGTCCTGAGTAA 3′)

### Data analysis

For each primer combination, fragment sizes in all samples were collected and repetitive bands were removed in Microsoft Excel. Total bands without repetition were obtained. Then, based on the total bands, a Perl script was compiled to transform the presence (1) or absence (0) of each band in an accession to binary data.

The genetic relationship was evaluated by a Bayesian approach using the software STRUCTURE 2.3.4 [36,37]. This revealed the genetic structure by assigning individuals or predefined groups to clusters. Ten runs of STRUCTURE were performed with the number of homogeneous genepools (*K*) from 1 to 10. Each run consisted of a burn-in period of 200,000 iterations followed by 200,000 Monte Carlo Markov Chain iterations, assuming an admixture model. The results were uploaded to the STRUCTURE HARVESTER web site [38] to estimate the most appropriate *K* value. Replicate cluster analyses of the same data resulted in several distinct estimated assignment coefficients, even though the same starting conditions were used. Therefore, we employed CLUMPP software [39] to average the 10 independent simulations and illustrated the result graphically using DISTRUCT [40].

A dendrogram was constructed based on Nei’s genetic distances [41] by the Neighbor-joining (NJ) method with 500 bootstrap replicates using TREECON (version 1.3b) [42].

## Results

### Polymorphisms of SSAP

The 93 pear accessions were analyzed for SSAPs using 12 primer combinations, which produced 2,833 fragments varying in size from 70 bp to 500 bp (S1 Table). A total of 2,799 fragments were polymorphic. The average percentage of polymorphic bands for all primer combinations was 98.80% (Table 3). For each primer combination, the number of bands was from 172 to 301. The polymorphic percentage of loci ranged from 0 to 100% (S1 Fig). The specificity increased with the band length (S1 Fig).

**Table 3:** The polymorphic characterization of sequence-specific amplification polymorphism primers in Asian pear.

Primer pairs	Number of bands	Number of polymorphic bands	Percentage of polymorphic bands
1 ppcr3/M-ACT	301	295	98.01
2 ppcr1/M-ACT	172	170	98.84
3 ppgr1/M-ACC	246	240	97.56
4 ppgr1/M-AGC	175	173	98.86
5 ppcr2/M-AAG	242	241	99.59
6 ppgr4/M-ACC	270	268	99.26
7 ppcr3/M-AAT	249	245	98.39
8 ppgr4/M-AGG	207	207	100.00
9 ppcr3/M-AAC	278	274	98.56
10 ppcr1/M-AGG	197	195	98.98
11 ppcr4/M-AAT	289	288	99.65
12 ppcr3/M-ACG	207	203	98.07
Total	2833	2799	98.80

### Genetic relationship of *Pyrus* accessions

**Fig 1:**
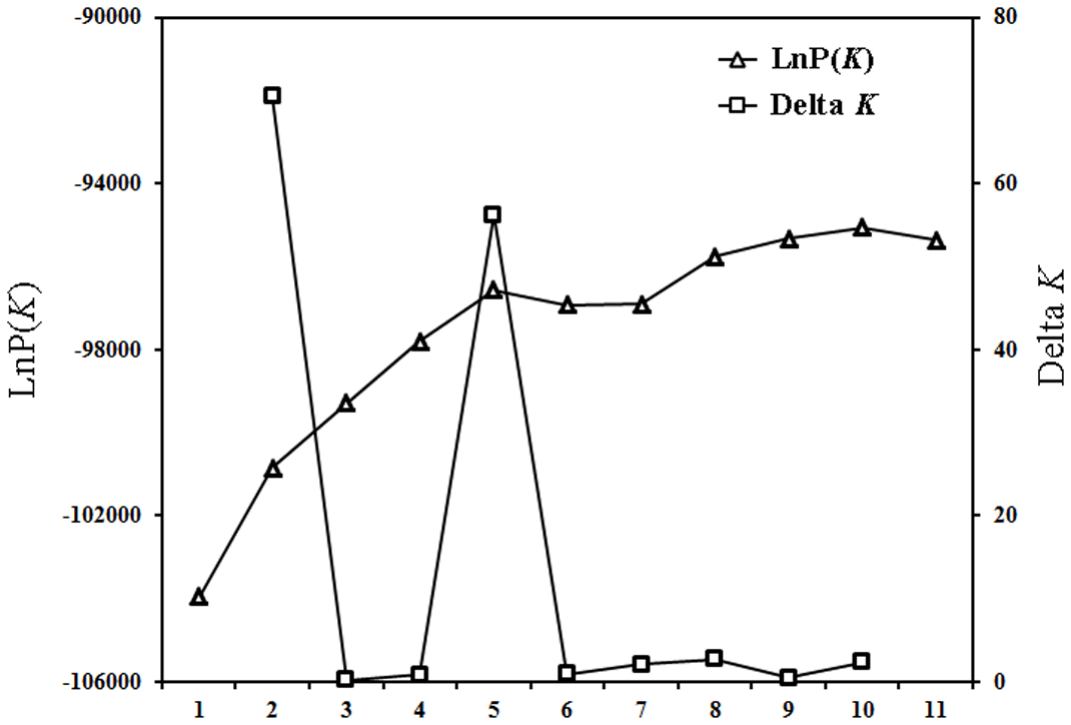
Modeling of cluster numbers for Asian pears using STRUCTURE software. LnP (*K*) and Delta *K* were calculated in accordance with the method of Evanno et al. [37].

**Fig 2:**
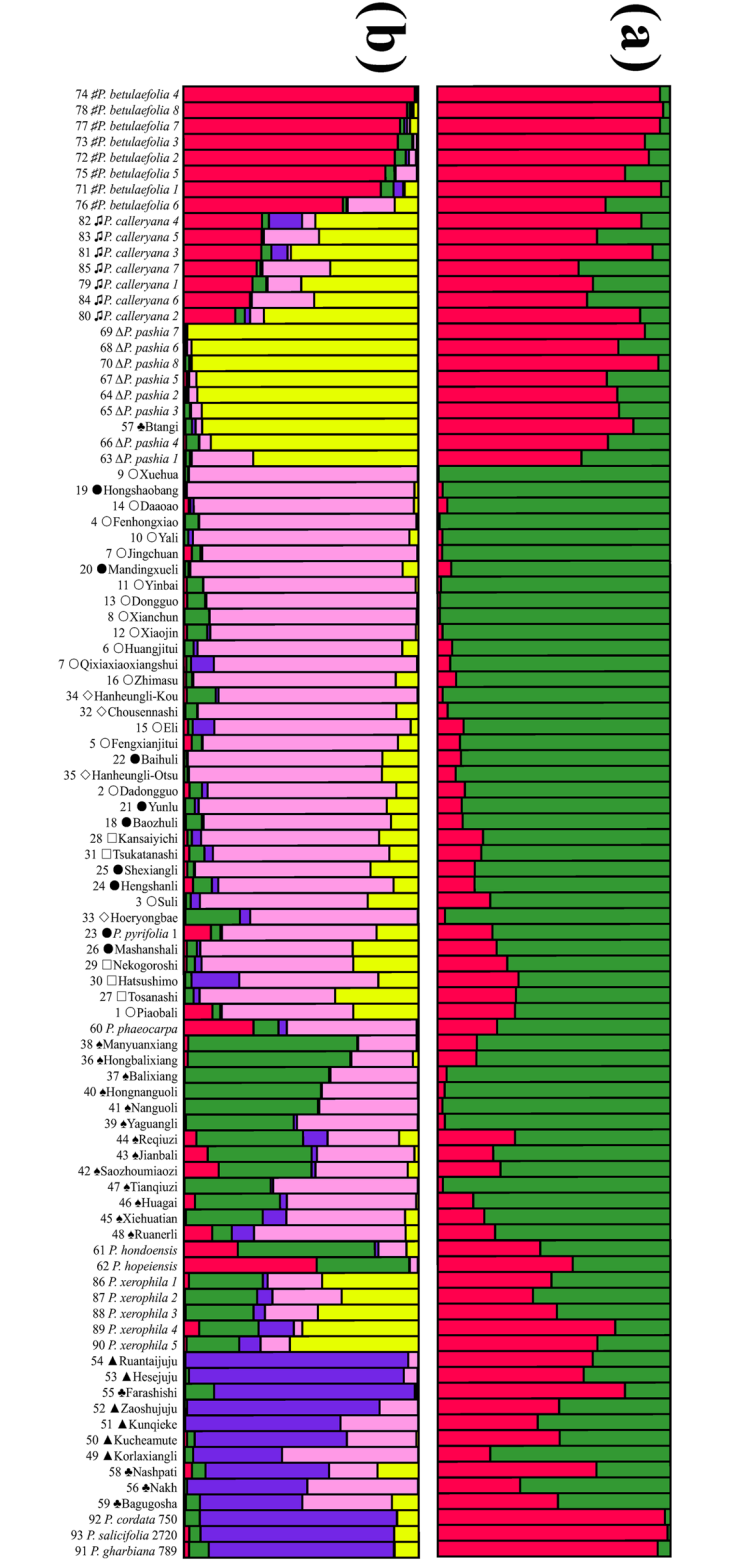
Genetic relationships among the 93 accessions of Asian pears revealed by a Bayesian modeling approach with number of genepools. (a) *K* = 2. (b) *K* = 5.

The number of homogeneous genepools (*K*) among the genotypes of the 93 accessions was modeled by Bayesian methods using the software STRUCTURE. The evaluation of the optimum number of *K*, which followed the procedure described by Evanno et al. [37], indicated two clear maxima for Δ*K* at *K* = 2 and 5 (Fig 1), suggesting that a model with two genepools captured a major split in the data, with substantial additional resolution provided under the model with *K* = 5. Barplots of the proportional allocations to each genepool for *K* = 2 and 5 in STRUCTURE are shown in Fig 2.

**Fig 3:**
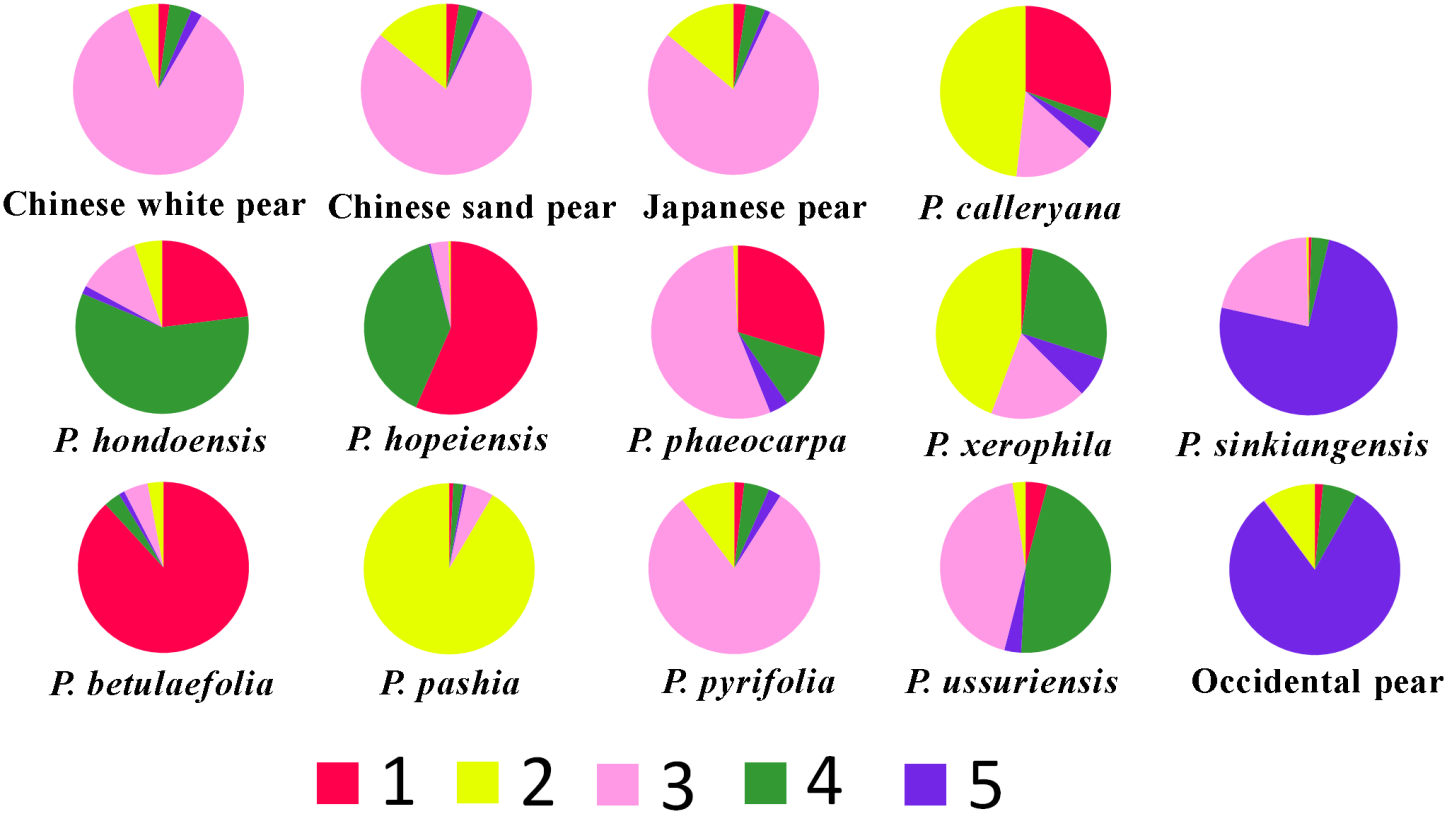
The composition of genepools (*K* = 5) in pear species/groups.

At *K* = 2, occidental pears and three wild Asian species (*P*. *pashia*, *P*. *calleryana*, and *P*. *betulaefolia*), constituted a genepool (red; Fig 2a). Most of the cultivars from Asia were in another genepool (green; Fig 2a). Under this model, the two genepools substantially overlapped, and the hierarchical levels in these two clusters could hardly be recognized. The model with five genepools was also strongly supported by the STRUCTURE result. Under this model, five genepools for all pear accessions were observed (Figs 2b and 3). The first genepool (red) consisted mainly of *P*. *betulaefolia* and *P*. *calleryana* accessions and a large portion of *P*. *phaeocarpa*, *P*. *hondoensis*, and *P*. *hopeiensis* clones were also in this genepool. The second genepool (yellow) consisted mostly of *P*. *pashia* and *P*. *calleryana* but also contained some accessions of *P*. *pyrifolia*, *P*. *xerophila*, and one accession of the Pakistani pear ‘Btangi’. Accessions of *P*. *calleryana* existed in several genepools, mainly in genepools one and two. The third genepool (pink) mainly consisted of *P*. *pyrifolia*, *P*. *ussuriensis*, and *P*. *sinkiangensis*. This genepool contained a large proportion of *P*. *pyrifolia* accessions. Two cultivars of ‘Xuehua’ and ‘Hongshaobang’ were present in a very high proportion in the third genepool. Some cultivars, such as ‘Mashanshali’, ‘Nekogoroshi’, ‘Tosanashi’, and ‘Piaobali’ were also present in a relatively large proportion in the second genepool. *P*. *ussuriensis* accessions were mainly in the third and fourth genepools (green). The green genepool also contained *P*. *xerophila*, *P*. *hondoensis*, and *P*. *hopeiensis*. The last genepool (blue) consisted mostly of occidental pears, Xinjiang pears (*P*. *sinkiangensis*), and majority of pears from Pakistan. In six accessions of *P*. *sinkiangensis*, two cultivars (‘Ruantaijuju’ and ‘Hesejuju’) existed in the last genepool. The other four cultivars of Xinjiang pears and accessions from Pakistan contained two major genepools, the third and fifth. For occidental pears, one major genepool (blue) was observed.

### The geographic distribution of genepools for pear accessions

**Fig 4:**
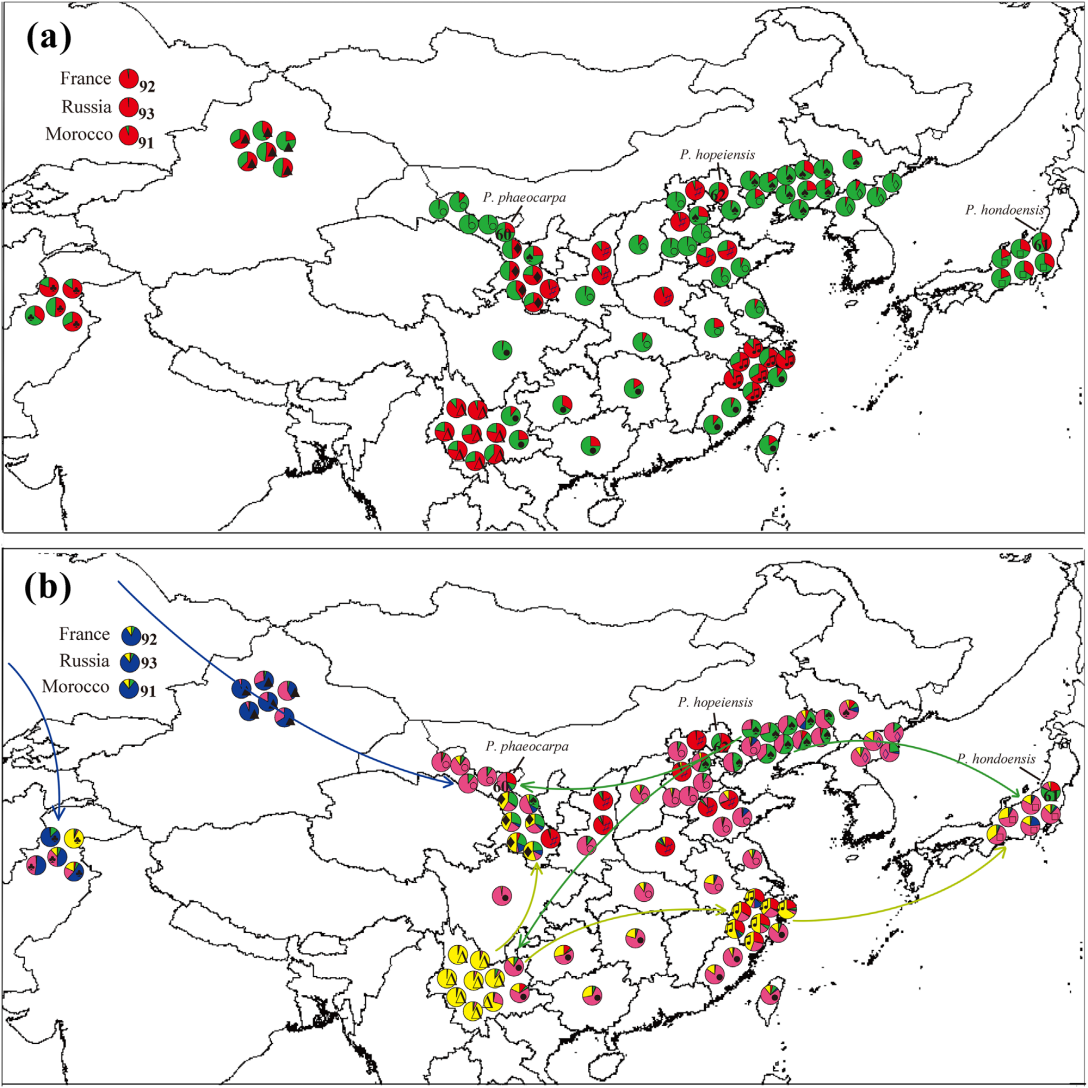
The geographic distribution of genepools of Asian pears. (a) Assignment of samples to two genepools under the cluster numbers *K* = 2 model. (b) Assignments of samples to five genepools under the *K* = 5 model. ‘○’: Chinese white pear; ‘●’: Chinese sand pear; ‘□’: Japanese pear; ‘◊’: Korean pear; ‘♠’: *Pyrus ussuriensis*; ‘▲’: *P*. *sinkiangensis*; ‘♣’: Pakistani pear; ‘Δ’: *P*. *pashia*; ‘♫’: *P*. *calleryana*; ‘♯’: *P*. *betulaefolia*; ‘♦’: *P*. *xerophila*. The three outgroup accessions are sited in the top left corner.

Under the model of *K* = 2, the two genepools were scattered in different regions (Fig 4a). The direction of genepool spread was hardly recognizable. Under the model of *K* = 5, the distribution of genepools was related to the species (Fig 4b). The third genepool (pink) was distributed extensively in Asia in accordance with the geographical distribution of *P*. *pyrifolia* (including Chinese white pear, Chinese sand pear, and Japanese pear). The last genepool (blue) mainly appeared in Xinjiang Province, Pakistan, and Europe where *P*. *sinkiangensis* and occidental pears exist (Fig 4b). The first genepool (red) was mainly distributed in northern China, which was related to the distribution of *P*. *betulaefolia*. The second genepool (yellow) appeared in southern China, northwestern China, and Japan, corresponding to the distribution of *P*. *pashia*, *P*. *pyrifolia*, and *P*. *xerophila*. The fourth genepool (green) was mainly in northeastern and northwestern China where *P*. *ussuriensis* and *P*. *xerophila* are distributed, and also in Japan where *P*. *hondoensis* (closer to *P*. *ussuriensis*) exists.

### Genetic relationships among *Pyrus* species and cultivars revealed by NJ dendrogram

Genetic relationships among the 93 pear accessions were revealed by neighbor-joining clustering approach. The NJ dendrogram (S2 Fig) clearly distinguished occidental pears from oriental pears. In oriental pear group, two wild pears groups were well supported including *P*. *pashia* (subgroup II) and *P*. *xerophila* (subgroup V). Accessions from *P*. *pyrifolia* (Chinese white pear and sand pear) and *P*. *ussuriensis* intermingled together, their relationship was poorly resolved. Japanese pears clustered together in Subgroup I with two cultivars from Korea and ‘Yunlu’, a Chinese sand pear from Zhejiang, China. Among the five Pakistan pear accessions, ‘Btangi’ fell into Subgroup II. ‘Nakh’ fell into Subgroup VI with the accessions of *P*. *sinkiangensis*, and the remaining three accessions clustered loosely between occidental and oriental pears in the dendrogram (S2 Fig).

## Discussion

### Characteristics of SSAP markers

In *Pyrus*, the most abundant retrotransposon families were *gypsy* and *copia*, constituting 25.5% and 16.9%, respectively, of the genome [27]. Although the role of retrotransposons in the genome evolution is not yet clear, considering that they comprise a large amount of the genome, they may be an essential part of the plant [30]. The amount of the genome consisting of retrotransposons varies among plants. In Rosaceae, 42.2% of the apple genome is retrotransposons [43], but in peach, this ratio decreased to 18.6% [43,44], which infers that retrotransposons have changed during the evolution of Rosaceae. In *Pyrus*, retrotransposon insertions indicated a high heterogeneity in different species [29]. Kim et al. [28] and Jiang et al. [29] developed RBIP markers, based on the oriental pear genome, that could be amplified in the oriental species of *Pyrus*. However, very few amplified in the occidental pear, suggesting that these retrotransposon insertions in the oriental pears occurred after the division of occidental and oriental pears. In this study, 12 SSAP primer combinations revealed high polymorphisms in different *Pyrus* species (Table 3, S1 Fig), which indicated that retrotransposons had replicated many times during pear development. The percentage of polymorphic bands identified by SSAP markers (98.8%) was higher than that of AFLP markers in *Pyrus* (89%, [16]), implying that the use of SSAP markers would be more efficient for resolving the genetic relationships among *Pyrus* species. Fluorescence primers were used in this study to analyze the band sizes, thus avoiding the errors of artificial counting and increased band relevance ratios. In this study, a total of 2,799 polymorphic fragments were obtained from the 12 primer combinations. The informative data from SSAP markers in pears was much more than those from AFLP (77 bands per primer combination, [16]) and SSR markers (28 bands per primer pair, [14]). Because of its low cost and simple protocols, fluorescent SSAP could be more widely used in studies on population structure and genetic relationships in plants.

### Primitive genepools and primary species in Asian pears

This is the first time to clarify a clear genetic relationship in *Pyrus* using retrotransposon-based markers. The STRUCTURE result (Fig 2) and NJ dendrogram (S2 Fig) divided *Pyrus* into oriental pears and occidental pears, which is in good accordance with the results obtained using AFLP, RAPD, and SSR markers, and DNA sequences [6,15,45].

The structure results provided two models for the genetic relationships of Asian pears. Under the model of *K* = 2, only two genepools (red and green) were identified (Fig 2a). Generally, the red genepool consisted of wild Asian species and occidental pears. The green genepool included all Asian cultivated pears and putative hybrid species. Although the model of *K* = 2 was supported by the structure (Fig 2a), it roughly separated wild and cultivated pears, but did not reveal much regarding the relationships among Asian pears. The model of *K* = 5 provided substantially more resolution (Fig 2b). Under this model, Asian pears, including putative hybrids, originated from five primitive genepools. Except for one genepool corresponded to the occidental pears, the remaining four primitive genepools corresponded to four Asian species: *P*. *pyrifolia*, *P*. *ussuriensis*, *P*. *betulaefolia*, and *P*. *pashia*. Therefore, these could be recognized as primary species as defined by Challice and Westwood [46]. The other species in Asia were included in at least two major genepools, which could be considered as hybrid origins.

### Gene introgression during cultivated pear development

Whether using the *K* = 2 or *K* = 5 structural model, the genotypes of almost all accessions were composed from at least two genepools, which implied that gene introgression commonly occurred during *Pyrus* evolution and the development of pear cultivars (Fig 2). This introgression was particularly apparent in cultivars of *P*. *pyrifolia* and *P*. *ussuriensis*. This finding supported the previous hypothesis that interspecific hybridization was the major mode of evolution in *Pyrus* [6,7,10,11].

There are three major cultivar groups in *P*. *pyrifolia*: Chinese sand pear, Chinese white pear and Japanese pear [3,9,16]. Japanese pears have been considered to be from the same germplasm as Chinese sand pears (*P*. *pyrifolia*). Recently, Iketani et al. [10] reported genetic differences between Japanese pear, Chinese pear, and Korean pear cultivars, and treated Japanese pears as a new cultivar group, named the *Pyrus* Japanese pear group. In NJ dendrogram (S2 Fig), five Japanese pear cultivars clustered in subgroup II with two Korean pears and one Chinese pear ‘Yunlu’ from Zhejiang Province. The result of STRUCTURE showed there was almost no difference between the Japanese pear and the Chinese sand pear (Figs 2b and 3). Although most Japanese pears clustered separately from Chinese accessions of the *P*. *pyrifolia* in a previous study, a few Japanese pears clustered closely with Chinese sand pears or white pears, especially those that originated from the coastal provinces of China, such as Zhejiang and Fujian [16]. Therefore, the primitive germplasm of the Japanese pear might have been introduced from ancient China, and then, Japanese pear cultivars developed independently, becoming specific.

Among the accessions of Chinese sand pears and white pears, two cultivars, ‘Xuehua’ and ‘Hongshaobang’, had very pure genepools (pink; Fig 2b), and other accessions had complex compositions of genepools, suggesting that most Chinese sand pears and white pears were of hybrid origin. The same situation also occurred in other cultivar groups or species, which explains the poor phylogenetic resolution of *Pyrus* mentioned in previous research [6,10]. In the past, we considered the current Chinese sand pear cultivars with large fruit as selected and developed artificially from the wild *P*. *pyrifolia* of the Changjiang River region. In this study, most accessions of *P*. *pyrifolia* were revealed to evolve by a process of introgressive hybridization between *P*. *pyrifolia* and *P*. *pashia*. The geographic distribution of *Pyrus* genepools indicated that Chinese sand pear cultivars in southwestern China and Japanese pears were from the same genepool (yellow), while Chinese white pear cultivars in northern China were from a different genepool (green; Fig 4b), which suggested that the gene introgression occurred from *P*. *pashia* to *P*. *pyrifolia* in southwestern China and Japan, while in northern China the gene introgression happened from *P*. *ussuriensis* to *P*. *pyrifolia*.

*Pyrus ussuriensis* is naturally distributed in Northeast China. A few studies revealed differences between cultivars and wild plants of *P*. *ussuriensis*, and introgression occurred among wild *P*. *ussuriensis* and cultivated *P*. *pyrifolia* [47,48]. In this study, the samples of wild *P*. *ussuriensis* were not included, but the genetic makeup of cultivars of *P*. *ussuriensis* could be clearly revealed. The results of the structure analysis showed that almost all of the *P*. *ussuriensis* cultivars were from two major genepools (Fig 2b), and one of them (pink) was from *P*. *pyrifolia*. It was noted that the *P*. *ussuriensis* cultivars had their own genepool (green) which inferred that the cultivars of *P*. *ussuriensis* should be involved in the hybridization between *P*. *pyrifolia* and wild *P*. *ussuriensis* which might have pure green genepool. The germplasm of *P*. *ussuriensis* cultivars were probably manipulated by human activity through hybridization with *P*. *pyrifolia* to produce a large fruit.

### Origins of hybrid species in Asia

The result of the genetic analysis showed clearly the origins of the putative hybrid species *P*. *calleryana*, *P*. *xerophila*, *P*. *sinkiangensis*, *P*. *phaeocarpa*, *P*. *hondoensis*, and *P*. *hopeiensis* from more than two genepools (Fig 3).

*Pyrus calleryana*, an important pea pear and excellent rootstock resource, was believed to be an ancestral *Pyrus* species [1,4] and was treated as a primary species [46]. However, in this study, *P*. *calleryana* occurred in two major genepools and a few minor genepools (Fig 3): one major genepool from *P*. *pashia* and the other genepool from *P*. *betulaefolia* (Fig 4b). *P*. *calleryana* and *P*. *betulaefolia* had some similar characteristics, including small fruit with (two or three) carpels and lobed leaves during the juvenile stages. However, the leaf shape and margin of *P*. *calleryana* were similar to those of *P*. *pashia*. According to the distribution of *Pyrus* [8], *P*. *betulaefolia* and *P*. *pashia* were both distributed in south part of Northwest China. The hybridization between *P*. *betulaefolia* and *P*. *pashia* occurred in the areas where these two species overlapped. Then, *P*. *calleryana* spread across other regions. A recent report based on multiple genes also indicated *P*. *calleryana* was polyphyletic [6].

*Pyrus xerophila* is mainly distributed in Gansu Province [8] and originated through ancient genetic recombinants that arose by interspecific hybridization involving both oriental and occidental species [6,29]. In our results (Fig 2b), this species was included in three major genepools (yellow, green, and pink/blue), indicating that the species originated through the hybridization of at least *P*. *pashia*, *P*. *ussuriensis*, and the occidental pears. Occidental pears may have been introduced into Gansu Province from central Asia and west Asia during ancient times through the Silk Road (Fig 4b) [13], which provides the possible hybridization origin of *P*. *xerophila*. *Pyrus sinkiangensis* was shown to contain hybrids between occidental pears and oriental pears [13]. In the results of the STRUCTURE (Fig 2b), two cultivars, ‘Ruantaijuju’ and ‘Hesejuju’, were from a major genepoo1 (blue), while the other four cultivars also contained another genepool (pink), which indicated the hybrid origin of *P*. *sinkiangensis* between occidental pears and *P*. *pyrifolia*, and the complex genetic composition within the species.

The origins of *P*. *phaeocarpa*, *P*. *hopeiensis* and *P*. *hondoensis* were revealed based on morphological characteristics [4,8] and DNA markers [9]. In this study, only single clones of *P*. *phaeocarpa*, *P*. *hopeiensis* and *P*. *hondoensis* were included. Therefore, remarks on their origins are subjective. However, genetic clues to the origins of these two species can be inferred. *P*. *phaeocarpa* shared two major genepools (pink and red; Fig 3) and one minor (green), which indicated that *P*. *phaeocarpa* might have originated from the hybridization of *P*. *betulaefolia*, *P*. *pyrifolia*, and *P*. *ussuriensis*. *P*. *hopeiensis* occurred in two major genepools (red and green) (Fig 3) which confirmed speculation that the hybrid origins of this species involved *P*. *betulaefolia* and wild *P*. *ussuriensis*. *P*. *hondoensis* contained elements of two major genepools (red and green) and two minor genepools (pink and yellow) (Figs 2 and 3), suggesting its close relationship with *P*. *ussuriensis* and the complex genetic composition resulting from hybridization with local species, most probably *P*. *dimorphophylla* Makino, which showed a close genetic relationship with *P*. *calleryana* [9] and was once classified as a variety of *P*. *calleryana*.

Five Pakistan accessions [49] were investigated in this study. ‘Btangi’ was identified as *P*. *pashia*, while ‘Nakh’ was grouped together with *P*. *sinkiangensis* (Fig 2, S2 Fig). This is the first report on the existence of *P*. *pashia* in Pakistan. The remaining three accessions were located between occidental and oriental pears in the NJ dendrogram (S2 Fig). In the STRUCTURE results (Fig 2b), these three species were in two genepools, that of the *P*. *pyrifolia* (pink) and that of the occidental pear (blue). We inferred that these three accessions were the result of hybridization events between occidental and oriental pears.

## Conclusion

This is the first study on the development of fluorescent SSAP markers based on retrotransposons for the evaluation of the genetic relationships in Asian pears. The result showed that all Asian pear species originated from five primitive genepools. The origin of two major cultivated pear species native to East Asia: *P*. *pyrifolia* and *P*. *ussuriensis* were involved in gene introgression. *P*. *calleryana*, *P*. *xerophila*, *P*. *sinkiangensis*, *P*. *phaeocarpa*, *P*. *hondoensis*, and *P*. *hopeiensis* were inferred to be hybrid species. Thus, fluorescent SSAP provided enough informative sites to efficiently reveal population structure and the complex hybrid origins in *Pyrus*. The method applied in this study provides a new insight into the origins of Asian pears and can be used in solving phylogenetic relationships of intrageneric taxa with complex hybrid origins.

## Supporting Information

S1 Fig The polymorphism of bands in each primer combination. (TIF)

S2 Fig Neighbor-joining tree of 93 pear accessions based on genetic distances as derived by Nei et al [41]. The numbers (1–93) represented code of accessions in sample list. ‘○’: Chinese white pear; ‘●’: Chinese sand pear; ‘□’: Japanese pear; ‘◊’: Korean pear; ‘♠’: *Pyrus ussuriensis*; ‘▲’: *P*. *sinkiangensis*; ‘♣’: Pakistani pear; ‘Δ’: P. pashia; ‘♫’: *P*. *calleryana*; ‘♯’: *P*. *betulaefolia*; ‘♦’: *P*. *xerophila*.
(TIF)

S1 Table Complete data set for 93 accessions amplified by 12 primer combinations. (XLSX)
