# Supplementary figures and images for: Primitive Genepools of Asian Pears and Their Complex Hybrid Origins Inferred from Fluorescent Sequence-Specific Amplification Polymorphism (SSAP) Markers Based on LTR Retrotransposons
Source: PLoS One. 2016 Feb 12;11(2):e0149192. doi: 10.1371/journal.pone.0149192 (PMC4752223; doi:10.1371/journal.pone.0149192)

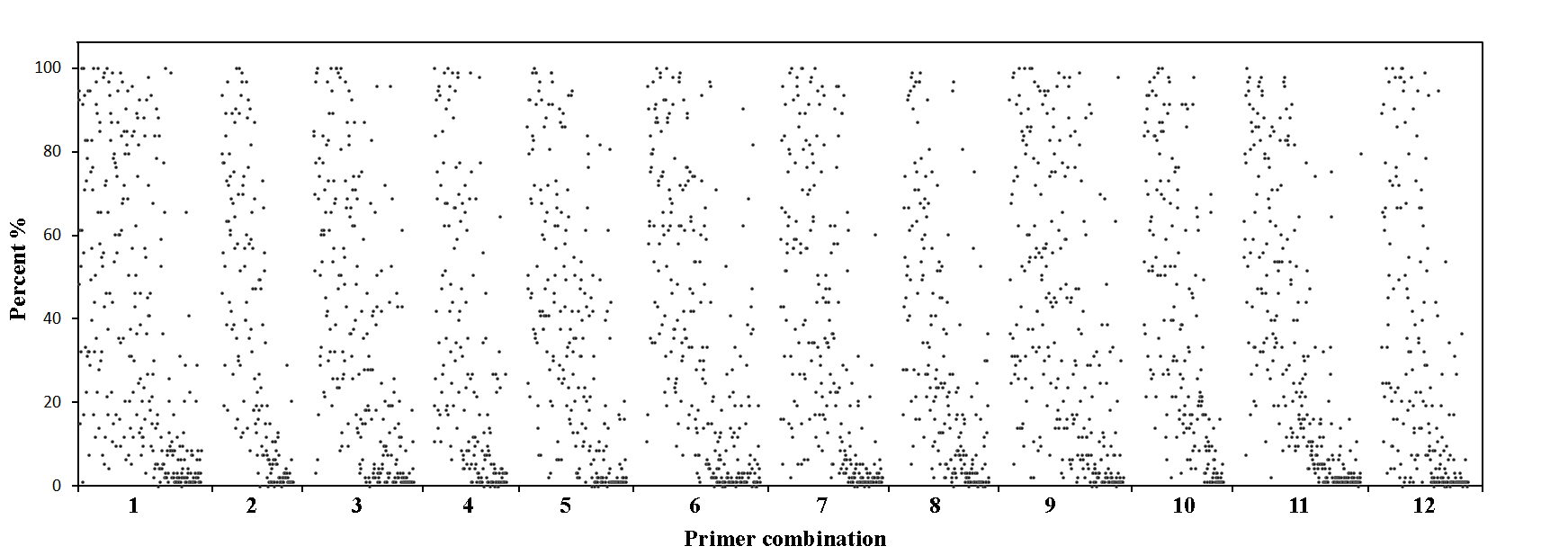

Supplement: S1 Fig — (TIF) [file pone.0149192.s001.tif]

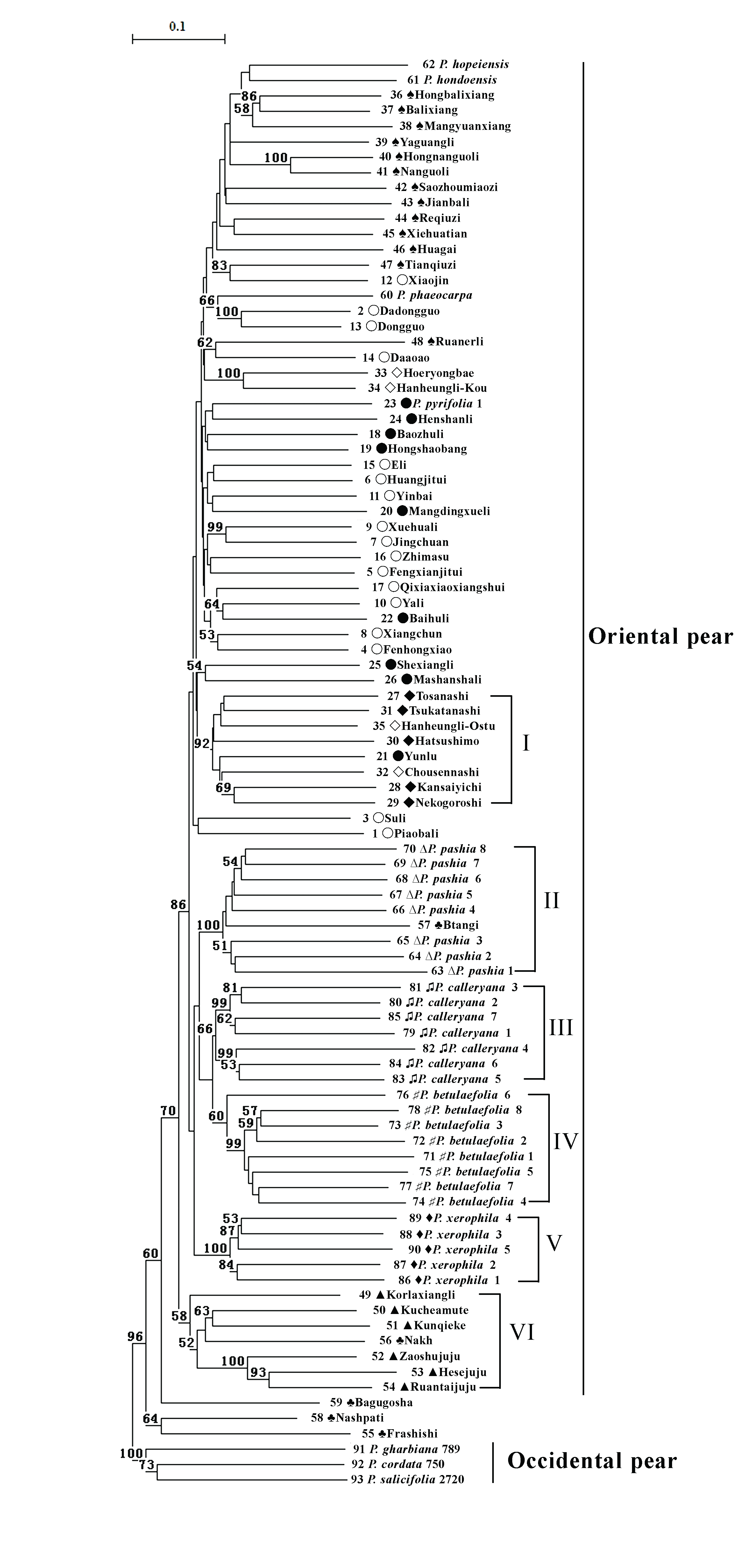

Supplement: S2 Fig — The numbers (1–93) represented code of accessions in sample list. ‘○’: Chinese white pear; ‘●’: Chinese sand pear; ‘□’: Japanese pear; ‘◊’: Korean pear; ‘♠’: Pyrus ussuriensis; ‘▲’: P. sinkiangensis; ‘♣’: Pakistani pear; ‘Δ’: P. pashia; ‘♫’: P. calleryana; ‘♯’: P. betulaefolia; ‘♦’: P. xerophila. (TIF) [file pone.0149192.s002.tif]
